# The appropriateness of prescribing antibiotics in the community in Europe: study design

**DOI:** 10.1186/1471-2334-11-293

**Published:** 2011-10-28

**Authors:** Evelien ME van Bijnen, Casper DJ den Heijer, W John Paget, Ellen E Stobberingh, Robert A Verheij, Cathrien A Bruggeman, Mike Pringle, Herman Goossens, François G Schellevis

**Affiliations:** 1 NIVEL, Netherlands Institute for Health Services Research, P.O. Box 1568, 3500 BN Utrecht, The Netherlands; 2 VU University Medical Centre, Dept. General Practice/EMGO+ Institute, Amsterdam, The Netherlands; 3 Department of Medical Microbiology, Maastricht University Medical Centre, School for Public Health and Primary Care (CAPHRI), The Netherlands; 4 University of Nottingham, Division of Primary Care, Nottingham, UK; 5 Vaccine and Infectious Disease Institute, University of Antwerp, Wilrijk, Belgium

## Abstract

**Background:**

Over 90% of all antibiotics in Europe are prescribed in primary care. It is important that antibiotics are prescribed that are likely to be effective; however, information about antibiotic resistance in the community is incomplete. The aim of our study is to investigate the appropriateness of antibiotic prescribing in primary care in Europe by collecting and combining patterns of antibiotic resistance patterns and antibiotic prescription patterns in primary care. We will also evaluate the appropriateness of national antibiotic prescription guidelines in relation to resistance patterns.

**Methods/Design:**

Antibiotic resistance will be studied in an opportunistic sample from the community in nine European countries. Resistance data will be collected by taking a nose swab of persons (N = 4,000 per country) visiting a primary care practice for a non-infectious disease. *Staphylococcus aureus *and *Streptococcus pneumoniae *will be isolated and tested for resistance to a range of antibiotics in one central laboratory. Data on antibiotic prescriptions over the past 5 years will be extracted from the electronic medical records of General Practitioners (GPs). The results of the study will include the prevalence and resistance data of the two species and 5 years of antibiotic prescription data in nine European countries.

The odds of receiving an effective antibiotic in each country will be calculated as a measure for the appropriateness of prescribing. Multilevel analysis will be used to assess the appropriateness of prescribing. Relevant treatment guidelines of the nine participating countries will be evaluated using a standardized instrument and related to the resistance patterns in that country.

**Discussion:**

This study will provide valuable and unique data concerning resistance patterns and prescription behaviour in primary care in nine European countries. It will provide evidence-based recommendations for antibiotic treatment guidelines that take resistance patterns into account which will be useful for both clinicians and policy makers. By improving antibiotic use we can move towards controlling the resistance problem globally.

## Background

Resistance to antibiotics is a growing public health problem [1-3]. The prevalence of antibiotic-resistant micro-organisms in both hospitals and the community is increasing [4-6]. Several studies have demonstrated that resistance frequently leads to a delay in the administration of effective therapy, which may be associated with increased costs, morbidity or even mortality [7,8].

A number of factors can explain the increasing trend in resistance but high exposure to antibiotics (which leads to a high selective pressure) is considered the most important cause [9]. Numerous individual and ecological studies have established a link between increased antibiotic consumption and the emergence of antibiotic resistance worldwide [10-12]. In Europe the most common exposure is the intake of antibiotic drugs, over 90% of which is prescribed in primary care. The variability of prescription rates is high: antibiotic use is low in northern, moderate in eastern and high in southern regions of Europe [6].

While the pharmaceutical industry is running out of options to develop new antibiotics, a way to decrease the exerted selective pressure is to cautiously and appropriately handle antibiotic prescriptions [13]. Antibacterial drug use has to be both necessary and appropriate to minimize the development of antibiotic resistance. It is unnecessary when no antibacterial drug is indicated and inappropriate when antibacterial treatment is indicated but an incorrect agent is selected (inactive against the most likely causative pathogen).

To assess the appropriateness of prescribing antibiotics in primary care, knowledge about likely aetiological agents and their resistance patterns is required [14]. When General Practitioners (GPs) are provided with data about the types and prevalence of resistant pathogens in their own region or country, antibiotic prescription could be optimised [15]. However, most resistance research has been carried out in hospital settings, and well-documented information about community resistance patterns is limited [4,16-19]. To support GPs in optimal antibiotic prescribing, it is necessary to define and encourage appropriate antibacterial use by utilising national data and developing evidence-based guidelines [20]. This study aims to fill this gap in knowledge [21], and will analyse the appropriateness of antibiotic prescribing in primary care. The main research question is: 'To what extent is the prescribing behaviour of primary care physicians in Europe congruent with the national or regional community antibiotic resistance patterns?'

Our analysis is twofold: Firstly, we will determine community resistance patterns in nine European countries and link these to the prescription behaviour of GPs to assess their congruency. We hereby hypothesize that higher antibiotic prescription rates are associated with higher resistance rates. Secondly, we will evaluate the current guidelines used by GPs with regard to the extent to which they are congruent with national resistance patterns, in order to make or revise recommendations, if necessary.

The resistance patterns and prescription patterns will be linked in this study to assess the appropriateness of prescribing antibiotics in Europe. We will specifically look at differences between countries, and as far as possible within countries. This article describes the design of this study and discusses the strengths and limitations of the study protocol. The results are expected at the end of 2013 and will be published in separate articles.

## Methods/Design

### Design overview

The research question will be answered using aggregated data in an ecological study design. We define the appropriateness of prescribing as the congruency between resistance patterns and prescription patterns; this will be operationalised as the odds that a patient will be given an effective drug. Data collection will be done within the APRES study (The appropriateness of prescribing antibiotics in primary care in Europe with respect to antibiotic resistance). The study will be conducted in nine countries in Europe: Austria, Belgium, Croatia, France, Hungary, the Netherlands, Spain, Sweden and the United Kingdom. We chose to include countries with different levels of prescription behaviour, to maximize our analysis range.

### Resistance data

#### Study population

##### a) Practices

GP practices (20 per country) are recruited through an existing GP network in each country [22]. This network should be representative of the GPs in that country or region as much as possible, and a prerequisite is the ability to deliver electronic prescription data of the participating practices for the past five years. Geographical spread will be obtained by recruiting practices in both rural as well as urban areas. Some countries (Spain, Croatia) will also include primary care paediatricians as these are the ones that deliver primary care to children in these countries.

##### b) Patients

The study will be carried out in the primary care setting and the target population is the community based without bacterial infections as a proxy for the general population. We use an opportunistic random sample of visitors of general practices. In the waiting room information leaflets and posters are used for recruitment purposes. All countries use the same inclusion criteria:

1) No antibiotic use in the past 3 months

2) No hospitalisation in the past 3 months

3) Registered patient or regular visitor of the practice for at least 1 year

4) Age 4 years and older (UK: 18 and over)

5) No residents of nursing homes

6) No existing infectious disease at time of visit for which antibiotics are prescribed

7) Not immunocompromised

8) No terminal illness

9) No out of hour consultations

During the period of data collection (November 2010 - May 2011) each practice recruits 200 patients to participate in the study. A Dutch study with the same design proved this to be feasible [23]. The data collection is spread across several months to maximize the potential detection of S. pneumoniae [24].

Whether a patient meets the inclusion criteria will be assessed by the GP or qualified practice nurse. Most criteria are apparent at first sight; others are asked when the patient is invited to participate. Regarding age and gender we strive for an equal stratification along the different groups; male, female and children (4-19), middle age (20-65) and elderly (> 65 years old). Recruitment will be monitored with a two-weekly update about the recruitment.

#### Resistance patterns

A nasal swab is taken from all participants by the GP or qualified practice nurse. The swabs are sent to one laboratory in each country using special envelopes. They should arrive within 48 hours, to increase the survival rate of *S. pneumoniae*. As a consequence of the ethical approval procedure, in the UK the nasal swabs are taken by the patients themselves, at home. All patients from whom a nasal swab was taken fill in a short questionnaire regarding their background and confounding variables. The national laboratories will isolate the bacteria (*Staphylococcus aureus *and *Streptococcus pneumoniae*) from the swabs (for the isolation procedures see additional file 1: Appendix A). These bacteria were selected because of their high impact on health care [25]. Prior to the swabbing period, the national laboratories are evaluated regarding the quality of their isolation procedures.

At the end of the data collection period, the isolated bacteria will be sent to the Department of Medical Microbiology of Maastricht University, the central laboratory of the project where the resistance testing will be performed. The antibiotic susceptibility of the isolated strains will be determined quantitatively using a broth dilution method in micro-titre plates according to the EUCAST standard [26]. The compounds tested include penicillins with and without beta-lactamase inhibitors, cephalosporines, macrolides, tetracyclines, quinolones, trimethoprim-sulfamethoxazole and rifampicin. For *S. aureus *also aminoglycosides and topical agents (fusidic acid and mupirocin) will be tested. This analysis will result in a resistance rate for every bacterium - antibiotic combination.

#### Sample size calculation

In a recent study among 2,000 community-dwelling persons in the Netherlands, 23% showed to be carrier of *Staphylococcus aureus*, whereas 4% showed to be resistant against antibiotics [23]. Power analysis showed that using a one-sided t-test (alpha = 0.05) testing for a 50 percent difference (4% versus 6%) between countries with a power of 0.95, 1,000 isolates are needed. For 1,000 isolates, 4,000 swabs have to be taken in each country. Therefore, for nine countries the total data collection will consist of 36,000 swabs.

### Prescription data

#### Study population

The GPs and GP practices who are participating in swabbing the patients also take part in the collection of prescription data.

#### Measurements

We will collect data about all prescribed antibiotics from the participating practices for the past 5 years (i.e. calendar years 2006 to 2011). For every prescription of antibiotics, i.e. antibacterials for systemic use according to the Anatomical Therapeutic Chemical (ATC) classification (ATC code J01), we will collect the following information:

• Date of the prescription;

• Diagnosis for which the antibiotic has been prescribed (if available);

• Identification of the chemical substance (7 digit ATC code);

• Number of packages (if available);

• Number of Defined Daily Dose (DDD's, if available).

The diagnostic data will be converted to the International Classification of Diseases,10th revision (ICD-10) [27]. No additional data collection is needed; all these data are routinely recorded in the patients' electronic medical records.

Data on characteristics of the practice population (the epidemiological denominator) including the size of the practice population and its age (in 5-year age bands) and sex distribution will also be collected. In countries with a registered list system, this file can be retrieved from the practice computer. In countries without a registered list system, estimates of the size and composition of the population will be provided [28] either based on characteristics of the patients visiting the practice, or based on extrapolation of local or regional population characteristics [29].

Finally, each practice will provide information on a few practice characteristics in a short questionnaire (geographical area, number of listed patients, number of physicians working in the practice, age of the physicians).

### Guidelines

In addition to the aforementioned data collection, relevant antibiotic treatment guidelines used in the nine participating countries will be collected and evaluated in relation to the resistance patterns in each country. In particular, we will focus on syndromes and diseases mainly caused by *S. aureus *and *S. pneumoniae*: skin infections and pneumonia respectively. In cooperation with the GP networks in the countries, we will assess the most frequently used primary care guidelines on both content (specific recommendations) and quality. For the latter we will use a standardised instrument, with a focus on the evidence base of the recommendations [30-33].

By comparing the current resistance patterns with the primary care guidelines per country, we will be able to make recommendations to improve these guidelines, incorporating the evidence we will collect.

### Ethical approval

In every participating country ethical approval for this study has been obtained. All participants sign an informed consent form. For children a separate form is developed, where consent from one of their parents or their guardian is obtained. Children are not allowed to participate in the UK due to ethical restrictions.

## Data analysis

Descriptive analyses will be carried out to calculate the resistance patterns and patterns of prescribed antibiotics in each country. For countries where diagnostic data are available, more in-depth analyses will be carried out to calculate the appropriateness of antibiotics prescribed for specific diseases. Gender and age specific analyses will be carried out if the sample size allows for meaningful analyses. In general, women, children and elderly have a higher health care utilisation than other groups, and therefore have a higher a priori risk of having antibiotics prescribed. Consequently, they may show higher rates of resistance and/or other resistance patterns. The frequency of prescribed antibiotics will be expressed as a rate per 1000 listed patients. The distribution of the different types of antibiotics will be calculated as proportions of all antibiotics prescribed (the 'market share').

To test our hypothesis and analyse the congruency of the resistance and prescription data we will calculate the odds of receiving an effective antibiotic on a national level. This 'effectiveness rate' will be calculated per country by multiplying the "market share" in prescriptions of a specific antibiotic with the established antibiotic resistance rate of the bacterium (e.g. if 50% of the prescribed antibiotics is penicillin and 20% of all bacteria is resistant to penicillin, penicillin's effectiveness rate is 50% × (100%-20%) = 40%). The effectiveness rate will be calculated per country for each combination of antibiotic and bacterium. The sum of the effectiveness rates is an indicator for the appropriateness of prescribing antibiotics in each country.

Since we use hierarchical data we will use a multilevel design to assess this appropriateness. Data are analysed with STATA and MLwiN for multilevel modelling. Potentially confounders will be included in the model to adjust for these variables.

## Discussion

### Strengths and limitations

This study will provide data from nine European countries on community based resistance patterns and antibiotic prescription patterns in primary care, thus assessing the appropriateness of prescribing antibiotics. Our integrated data set with a range in prescription and resistance patterns is powerful and fills the gap in knowledge concerning community resistance patterns. We will not only provide information on resistance patterns, but also relate them to prescription behaviour and treatment guidelines. To improve the quality of prescribing it is important to monitor antimicrobial resistance and usage data on a national level and benchmark these by comparisons with other countries [34].

Our first intent was to measure resistance in a random sample of the general population. Due to practical reasons this proved not to be feasible. The opportunistic sampling method that was chosen instead is potentially prone to selection bias. With our stratification methods we will be able to reduce this bias and be able to conduct analyses concerning several age groups. In the UK, we are using a different design, where patients swab themselves. This may lead to a specific selection of patients. However, since we also measure background variables we are able to compare the participants' characteristics with those in other countries. Also, since we only measure at one point in time we cannot indicate a causal relationship between prescription behaviour and resistance patterns. However, our study will produce valuable data on a public health level.

### Implications

Our consistent design throughout Europe provides an important insight into the appropriateness of prescribing antibiotics. With our results, we will be able to make evidence-based recommendations for antibiotic treatment guidelines. Clinicians and policy makers can both profit from this knowledge. By improving antibiotic use and decreasing resistance at local levels, we can move towards controlling the resistance problem in Europe. It would be valuable to continue monitoring resistance patterns in the community in the future.

## Competing interests

The authors declare that they have no competing interests.

## Authors' contributions

EvB has drafted the manuscript; JP, CdH, CB, FS and RV have critically reviewed it. ES, MP, HG, RV and FS designed the study and obtained funding. All authors read and approved the final version of the manuscript.

## Pre-publication history

The pre-publication history for this paper can be accessed here:

http://www.biomedcentral.com/1471-2334/11/293/prepub

## Supplementary Material

Additional file 1 **Laboratory Protocol**. Laboratory Protocol for isolating S. aureus and S. pneumonia. Click here for file
