# Supplementary material for: The appropriateness of prescribing antibiotics in the community in Europe: study design
Source: BMC Infect Dis. 2011 Oct 28;11:293. doi: 10.1186/1471-2334-11-293 (PMC3220649; doi:10.1186/1471-2334-11-293)
Supplement: Additional file 1 — Laboratory Protocol. Laboratory Protocol for isolating S. aureus and S. pneumonia. [file 1471-2334-11-293-S1.DOC]

**Appendix A: Laboratory Protocol for isolating *S. aureus* and *S. pneumoniae***

| Micro-organism | Preparation swab | Incubation | Morphology | Identification tests | Storage isolates  in skimmed milk cryovials |
| --- | --- | --- | --- | --- | --- |
| *S. aureus* | Plate nasal swab on  standard blood agar  plate  AND  selective agar plate (blood agar + gentamicin) | For 18-24 hours at 35^o^C in a  CO_2_-enriched atmosphere | Small to middle sized (β-hemolytic) white-yellow colonies, | Coagulase  (clumping/ tube)  Or  Latex agglutination | In duplicate |
| *S. pneumoniae* | Plate nasal swab on  standard blood agar  plate  AND  selective agar plate (blood agar + gentamicin) | For 18-24 hours at 35^o^C in a  CO_2_-enriched atmosphere | Small α-hemolytic colonies; umbilicate, transparent, flattened or teardrop-shaped (characteristic central depression) | Optochin  In case of doubt:  Bile esculin/solubility  or API  or VITEK | In duplicate |
